# Study on the Association between Tail Lesion Score, Cold Carcass Weight, and Viscera Condemnations in Slaughter Pigs

**DOI:** 10.3389/fvets.2016.00024

**Published:** 2016-03-14

**Authors:** Dayane Lemos Teixeira, Sarah Harley, Alison Hanlon, Niamh Elizabeth O’Connell, Simon John More, Edgar Garcia Manzanilla, Laura Ann Boyle

**Affiliations:** ^1^Pig Development Department, Teagasc Animal and Grassland Research and Innovation Centre, Moorepark, Ireland; ^2^Departamento de Ciencias Animales, Pontificia Universidad Católica de Chile, Santiago, Chile; ^3^UCD School of Veterinary Medicine, University College Dublin, Belfield, Ireland; ^4^Institute for Global Food Security, Northern Ireland Technology Centre, Queens University Belfast, Belfast, UK

**Keywords:** carcass, condemnation, meat inspection, pig, tail lesion, viscera

## Abstract

The aim of this study was to assess the relationship between tail lesions, cold carcass weight, and viscera condemnations in an Irish abattoir. The following data were collected at the evisceration point from every third pig slaughtered over 7 days: farm identification, sex, tail lesion score, viscera inspection outcome, and cold carcass weight. Tail lesions were scored according to a 5-point scale. Disease lesions responsible for lung (pleurisy, pneumonia, and abscess), heart (pericarditis), and liver (ascariasis) condemnation were recorded based on the decision of the veterinary inspector (VI). Data on 3,143 pigs from 61 batches were available. The relationship between disease lesions, tail lesion score, and cold carcass weight was studied at individual carcass level, while the relationship between disease lesions and tail lesion score was studied at both carcass and batch level. Tail lesions (score ≥1) were found in 72% of the study population, with 2.3% affected by severe tail lesions (scores ≥3). Pleurisy (13.7%) followed by pneumonia (10.4%) showed the highest prevalence, whereas the prevalence of ascariasis showed the greatest variation between batches (0–75%). Tail lesion score, pleurisy, pleuropneumonia, and pericarditis were associated with reductions in carcass cold weight (*P* ≤ 0.05) ranging from 3 to 6.6 kg. Tail lesion score was associated with condemnations for pleurisy, pneumonia, and pleuropneumonia (*P* ≤ 0.05) at a batch level. VI shift was associated with condemnations for pneumonia, pleuropneumonia, and pericarditis (*P* ≤ 0.05) at a carcass level and with pneumonia at a batch level. Sex was not associated with viscera condemnations but males were more likely to be affected by tail lesions. The relationship between overall tail lesion score and the lung diseases at batch level supports the relationship between poor health and poor welfare of pigs on farms. The inclusion of tail lesion scores at post-mortem meat inspection should be considered as a health and welfare diagnostic tool.

## Introduction

The primary function of meat inspection is the protection of public health (1). However, there is considerable variation internationally in the amount and quality of data relating to animal health, which are routinely collected at meat inspection (2). Some data are routinely collected during meat inspection on carcass condemnation (3, 4), but little are available with primary relevance to animal health or welfare (4).

In some countries, data are routinely collected at meat inspection for disease surveillance (1) as well as for tracing affected herds in national disease control programs [e.g., *Salmonella*; Alban et al. (5)]. These data are used in epidemiological studies of disease to investigate risk factors (6), geographical or seasonal differences (3), and variations between herds (7). There is growing interest in the collection of information relating to animal welfare at meat inspection (8–10). Abattoir meat inspection has several advantages over farm-based inspections for the collection of data relating to animal-based welfare outcomes (11). EFSA (12) described many animal-based welfare outcomes that can be measured ante-mortem or post-mortem during meat inspection. Of these, tail lesions are of particular relevance to pig welfare. Tail biting is a widespread behavioral vice of pigs, resulting in poor performance and carcass condemnation (13). The problem also reflects deficiencies in the pigs’ environment and health status (14). Tail lesions have potential as “iceberg” indicators of pig health and welfare on farm (15). Furthermore, they are highly prevalent and easy to detect and score at meat inspection (2, 16).

Tail damage may provide routes for the spread of infection (17). This explains the association of tail damage with certain pathological lesions (17, 18). Indeed, the relationship between abscessation and tail biting is particularly well-documented (16, 19–22). However, the association between tail damage and other lesions may also be explained by shared risk factors (23–25). It is worth noting that even in the absence of overt tail biting, there may also be a high prevalence of persistent tail chewing and tail manipulatory behaviors performed by pigs in commercial systems (26). Evidence suggests that even mild tail damage restricted to puncture wounds can readily set up pyaemia (27) and is also associated with lighter carcass weights (16). Tail lesions are also associated with lung pathologies, such as pneumonia, abscesses, and pleuritic lesions in the lungs (3, 21, 22). Ultimately, such pathologies may lead to lung condemnations (28).

The relationship between tail biting and diseases or condemnations of the heart and liver are less well investigated. Nevertheless tail lesion severity scores have potential to be used as a predictor for the presence of internal lesions (21). Therefore, the main aim of this study was to evaluate the relationship between tail lesions and viscera condemnations. Ultimately, the existence of such a relationship could help strengthen the case for the inclusion of tail lesion severity scoring in the meat inspection process. The data presented in this manuscript are based on observations that were partly published in Harley et al. (16). In that study, associations between tail lesion scores and cold carcass weights were found. Hence, a secondary aim of this work was to determine relationships between tail and disease lesions responsible for viscera condemnations and cold carcass weights.

## Materials and Methods

### Data Collection

The study was conducted over 7 days during April 2012 in an Irish abattoir, with a weekly throughput of approximately 10,500 pigs. The sample size calculation was generated using data from the literature (20) and AusVet Epitools software (29), as described in detail by Harley et al. (16). Data were collected at three points on the slaughter line: (I) between dehairing and evisceration; (II) at post-mortem meat inspection; and (III) at the weighing scales. A sampling interval of every third pig was used.

**Figure 1:**
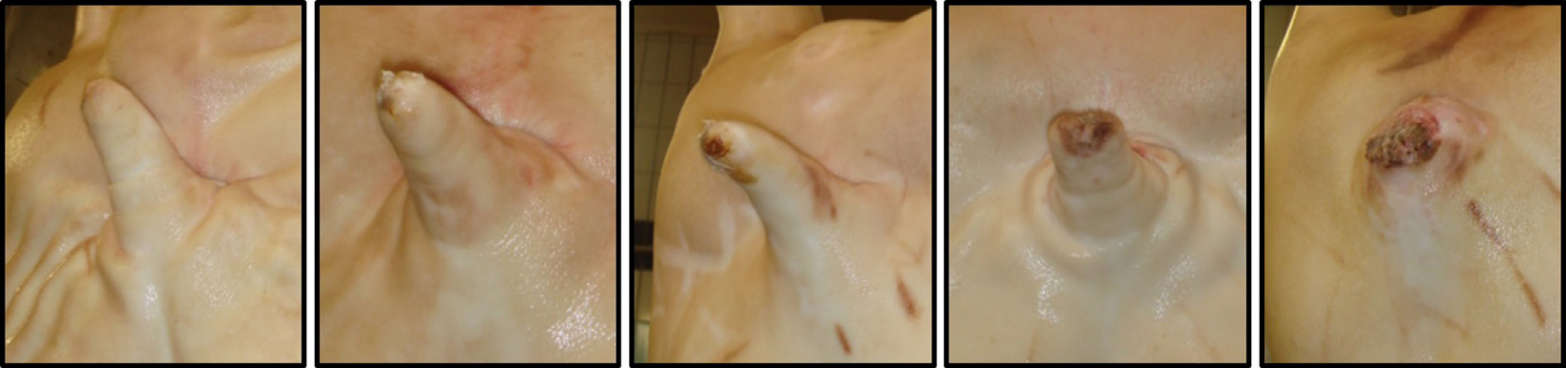
**Tail lesion scoring system adapted from Kritas and Morrison (21) (Scores 0–4, left to right)**. (0) No evidence of tail biting. (1) Healed or mild lesions. (2) Evidence of chewing or puncture wounds, but no evidence of swelling. (3) Evidence of chewing or puncture wounds with swelling and signs of possible infection. (4) Evidence of chewing or puncture wounds with severe swelling/infection or open, gaping wound in cases of complete tail amputation.

At the first data collection point, an identification tag was suspended from one hind foot of each study carcass, sex and herd identification codes were recorded (further classified as batch), and tail lesions were scored on a 0–4 scale (Figure 1). The same person scored tail lesions throughout the study. At the second data collection point, the reason and anatomical locations of carcass condemnations and trimmings [data defined and presented in Harley et al. (16)] and disease lesions responsible for lung (pleurisy, pneumonia, pleuropneumonia, and abscess), heart (pericarditis and endocarditis), and liver (ascariasis) condemnation (Table 1) were recorded as present or absent by the same person throughout the experiment. These records were on the basis of the decision of the acting Department of Agriculture, Food and the Marine (DAFM) temporary veterinary inspector(s) (VI) on the line. All data were collected from 0900 hours to approximately 1800 hours. There were three VI shifts (VIS), each of three people, working separate shifts to the following daily schedule: shift 1, 0700–1030 hours; shift 2, 1050–1420 hours; and shift 3, 1450–1750 hours. For the majority of shifts, the VI teams included the same individuals; however, there were some substitutions during the study. VIS 1 scored 666 carcasses from 10 farms and 13 batches; VIS 2 scored 1484 carcasses from 19 farms and 27 batches; and VIS 3 scored 993 carcasses from 17 farms and 21 batches. At the third data collection point, one person removed the identification tag from the hind foot and recorded the line “kill number” of the study in order to later obtain cold carcass weights.

**Table 1:** **Definition of diseases associated with viscera (lungs, heart, and liver) condemnations detected at meat inspection**.

Anatomy affected	Disease	Appearance/description
Lungs	Pleurisy	Fibrotic adhesions to thoracic wall
		Fibrosis of pleural membrane
	Pneumonia	Plum-colored consolidation of lung parenchyma (generally in cranio-ventral lobes)
	Abscessation	Focal, circular, encapsulated yellow–green lesion protruding from surface of lung parenchyma
Heart	Pericarditis	Fibrosis of pericardial sac, with or without fluid
	Endocarditis	Inflammation of the inner layer of the heart, the endocardium
Liver	Ascariasis	Multifocal fibrotic lesions in liver stroma, the so-called “milk spot liver” resultant from *Ascaris suum infection*

*Source: Ref. (28)*.

### Statistical Analysis

Descriptive statistics were calculated using Microsoft^®^ Excel^®^ for Windows and all other statistical analyses were conducted using SAS 9.3. Dependent variables to be studied were disease lesions and carcass weight. Disease lesions responsible for viscera condemnations were classified as present or absent, with the latter including only carcasses without any other disease lesion. Carcasses with two different types of lesions responsible for viscera condemnation were excluded from the statistical analyses (except for carcasses with both pleurisy and pneumonia that formed an extra category of disease lesions). Due to the low prevalence of endocarditis, it was not considered for statistical analysis. Explanatory variables were sex, VIS, and tail lesion score. Disease lesions were also considered as explanatory variables for cold carcass weight. Tail lesion scores 3 and 4 were combined due to the low number of cases in both categories.

Data were analyzed at two levels, carcass level (for disease lesions and carcass weight) and batch level (for disease lesions). For farms that sent batches on different days, each batch was considered separately. Batches with <20 pigs recorded were excluded from the data set.

The analysis of the association of carcass weight with explanatory variables was conducted using general linear mixed models (Proc mixed), with inclusion of all second-order interactions and batch as a random effect. Carcasses condemned or trimmed were not included in this analysis. Carcass weight was first studied in a bivariate analysis for each factor and then in a multivariable model. A colinearity effect between sex and tail lesion score was found and sex was not included in the final model as the variable having the lowest association with the dependent variable.

The association of disease lesions with sex, VIS, and tail lesion score at a carcass level was studied using generalized linear mixed models (Proc glimmix), including batch as a random effect. Each dependent variable was first studied in a bivariate analysis for each factor and then a multivariable model was done. A colinearity effect between sex and tail lesion score was found; therefore, sex was not included in the final model.

The association of disease lesions with VIS and tail lesion score at a batch level was also studied using generalized linear mixed models (Proc glimmix). For this model, an overall tail lesion score was calculated for each batch by weighting the tail scores (i.e., proportion of carcasses with score 1 × 1 + proportion of carcasses with score 2 × 2 + proportion of carcasses with score 3 × 3 + proportion of carcasses with score 4 × 4).

In all the models, alpha level for determination of significance was 0.05. Tendencies toward significance were presented for alpha 0.05–0.10. Data are presented as least square means ± SEs.

## Results

### Descriptive Results

A total of 3537 pigs were observed during the study. Batches with <20 pigs were excluded and, consequently, the final study population included 3143 pigs from 61 batches and 36 farms. Ten carcasses were fully condemned and 62 were partially condemned, while 102 were trimmed. The reason and location of partial condemnations and trimmings are described in detail in Harley et al. (16).

The carcass-level prevalence of tail lesions and disease lesions responsible for viscera condemnation are shown in Table 2. Tail lesions (score ≥1) were found in 72% of the study population, with 2.3% affected by severe tail lesions (scores ≥3) and more males affected by scores of ≥1 than females. A total of 1114 cases of disease lesions responsible for viscera condemnation were recorded, with approximately 71% being related to lung diseases, 8% related to heart disease, and 16% related to liver disease. A total of 61 carcasses had the lungs condemned for both pleurisy and pneumonia.

**Table 2:** **General description of the study animals, including the percentage of tail lesions and disease lesions associated with viscera condemnation**.

	Female	%	Male	%	Total	%
Farms	–	–	–	–	36	–
Batches	–	–	–	–	61	–
Pigs	1526	48.5	1617	51.5	3143	100.0
**Tail lesions**						
Score 0	510	33.4	372	23.0	882	28.1
Score 1	681	44.6	771	47.7	1452	46.2
Score 2	316	20.7	421	26.0	737	23.4
Score 3	12	0.8	30	1.9	42	1.3
Score 4	7	0.5	23	1.4	30	1.0
**Diseases associated with viscera condemnation**						
Lung						
Pleurisy	213	14.0	223	13.8	436	13.9
Pneumonia	146	9.6	195	12.1	341	10.9
Pleuropneumonia	29	1.9	32	2.0	61	1.9
Abscessation	6	0.4	6	0.4	12	0.4
Heart						
Pericarditis	38	2.5	48	3.0	86	2.7
Liver						
Ascariasis	100	6.6	78	4.8	178	5.7

Batch-level descriptive data, relating to overall tail lesion scores, cold carcass weights, and the prevalence of the disease lesions responsible for viscera condemnation at batch level, are shown in Table 3. As the batches with <20 pigs were excluded from the dataset, batch size ranged from 20 to 108 pigs, with an average of 51.5 ± 20.90 pigs per batch. At least one batch presented 0% for each of the disease lesions responsible for viscera condemnation. Pleurisy (13.7%) followed by pneumonia (10.4%) showed the highest prevalence but the prevalence of ascariasis showed the greatest variation between batches (0–75%).

**Table 3:** **Description of the study batches (mean values and SD, minimum and maximum values), including batch size, overall tail lesion score, cold carcass weight, and disease lesions responsible for viscera condemnation within batches**.

	Mean	SD	Minimum	Maximum
Batch size (pigs)	51.5	20.90	20.0	108.0
Overall tail lesion score	1.0	0.42	0.2	2.1
Cold carcass weight	79.6	4.69	63.0	89.8
**Disease lesions responsible for viscera condemnation^a^**				
Lung				
Pleurisy	13.7	13.42	0.0	61.1
Pneumonia	10.4	12.18	0.0	55.8
Pleuropneumonia	1.7	3.65	0.0	20.6
Abscessation	0.3	0.97	0.0	7.1
Heart				
Pericarditis	2.8	3.94	0.0	22.2
Liver				
Ascariasis	7.3	15.60	0.0	75.0

*^a^Prevalence within batch*.

### Cold Carcass Weight Results

The association between disease lesions responsible for viscera condemnations and carcass cold weight at individual carcass level are presented in Table 4. Scores 2, 3, and 4 were grouped due to the low number of carcasses affected. There was a significant negative effect of tail lesion severity score on the cold weight of carcasses without a disease lesion responsible for viscera condemnation. Carcasses without any viscera condemnations and moderate or severe tail lesions (score ≥2) were 1.3 kg lighter than those with mild tail lesion scores (score 1; *P* ≤ 0.05).

**Table 4:** **Least square means ± SE (number of pigs in each category) of cold carcass weight (kg) with disease lesions responsible for viscera condemnation (not including carcasses condemned and/or trimmed) within tail lesion score**.

Reason for viscera condemnation	No. of carcasses*	0	1	≥2
No disease	2096	80.3 ± 0.65 (634)^A,B,a^	80.9 ± 0.62 (985)^A,a^	79.6 ± 0.71 (477)^B,a^
Lung				
Pleurisy	319	76.8 ± 1.11 (77)^b^	77.9 ± 0.91 (150)^b^	77.1 ± 1.08 (92)^b^
Pneumonia	250	77.8 ± 1. 22 (58)^a^	79.6 ± 1.02 (120)^a^	78.4 ± 1.24 (72)^a^
Pleuropneumonia	55	73.7 ± 2.96 (10)^b^	76.7 ± 1.90 (24)^b^	75.7 ± 2.18 (21)^a^
Heart				
Pericarditis	42	75.7 ± 2.50 (11)^a^	76.1 ± 1.83 (20)^b^	81.8 ± 2.57 (11)^a^
Liver				
Ascariasis	134	82.1 ± 1.60 (33)^a^	83.2 ± 1.34 (58)^a^	83.0 ± 1.54 (43)^b^

*Lung abscessation was not included due the low number of carcasses affected*.

*Number of carcasses that presented only each disease lesion. These data do not include carcasses condemned and/or trimmed and from batches smaller than 20 carcasses.

*^A,B,C^With different uppercase superscripts indicate significant differences within rows*.

*^a,b^with different lowercase superscripts indicate significant differences between the condition of viscera condemnation (yes or no) within tail lesion score*.

Within each disease lesion, carcasses had similar weights independent of the tail lesion severity (*P* ≥ 0.05).

Carcasses with tail lesions scored as none or mild (score ≤1) and lungs condemned for pleurisy and pleuropneumonia were lighter than those with no viscera condemned and similar tail lesion scores (*P* ≤ 0.05). Carcasses with tail lesions scored as mild (score 1) and that had the heart condemned for pericarditis were also lighter than those with no viscera condemned and similar tail lesion scores (*P* ≤ 0.05).

Carcasses with tail lesions scored as moderate or severe (score ≥2) and for which viscera were condemned for pleurisy and ascariasis were 2.5 kg lighter and 3.6 kg heavier, respectively, than those with no viscera condemned and similar tail lesion scores (*P* ≤ 0.05).

### Disease Lesions Results

At the level of the individual carcass, tail lesion score had no relationship with the disease lesions (*P* > 0.05). VIS showed an effect on pleuropneumonia (*P* = 0.018) and pericarditis (*P* = 0.004). Sex was not associated with any of the reasons for viscera condemnation in the bivariate analysis with batch as a random factor. However, sex was clearly related to tail score (*P* < 0.001) and was removed from the multivariable model to avoid colinearity.

For data analyzed at a batch level, there were relationships between the disease lesions responsible for viscera condemnation, VIS, and overall tail lesion. VIS was associated with condemnations for pneumonia (*P* < 0.001) and tail lesion score was associated with condemnations for pleurisy (*P* = 0.035), pneumonia (*P* = 0.004), and pleuropneumonia (*P* = 0.021). There was also an interaction between the effect of VIS and tail lesion score on condemnations for pneumonia (*P* < 0.001).

## Discussion

### Descriptive Results

Routine tail docking is no longer permitted under EU Council Directive 2008/120/EC, but the effectiveness of this method as a control for tail biting is widely discussed (30). While docking clearly reduces the risk of tail biting (31), it does not eliminate it (30). Consistent with this, although almost 100% of Irish pigs are docked (2), a high prevalence of tail lesions (72%) was detected in this study.

The prevalence of the lung diseases (pleurisy, pneumonia, and abscesses) and that of livers affected by white spots were higher than the mean prevalence reported by Elbers et al. (3) and Tuovinen et al. (32). However, it is difficult to compare such data between studies because of the numerous sources of variation that exist and which influence the effectiveness of detecting clinical signs of diseases (11). These include variation between people in detecting disease conditions (11), line-speed, intensity of working conditions and recording methods employed (3, 33), or even the variation in the description of identical conditions and terminology (3). Nevertheless, the high prevalence of these conditions recorded could be considered a cause for concern for the health of the Irish national pig herd.

### Effect of Disease Lesions and Tail Lesions on Cold Carcass Weight within Tail Lesion Score

Tail biting represents an important source of financial loss because it is associated with a reduction in animal performance (34). Pigs with severe tail lesion scores have low weight gain (22, 35) and lighter cold carcass weights (16). Similarly, sick pigs are lighter (36) as many diseases cause discomfort and lower feed intake resulting in lower growth rates and/or an increase in the number of days to slaughter and ultimately lighter carcasses.

Harley et al. (16) already reported a negative effect of tail lesion severity score on cold carcass weight on the same set of carcasses as used in the current study. The present study expands these findings by showing a difference in cold carcass weight depending on the presence or absence of a viscera disease lesion leading to condemnation. For carcasses without any disease lesions, there was an average reduction in weight of 1.3 kg associated with tail lesions scored as moderate or severe (scores ≥2) relative to tails scored 1. At the same time, within carcasses with tail scores of 0 or 1, pleurisy, pleuropneumonia, and pericarditis were associated with reductions in cold carcass weight ranging from 3 to 6.6 kg when compared to unaffected pigs. Thus, reductions in cold carcass weight associated with respiratory disease are similar to those observed for moderate or severe tail lesion scores.

Within tail lesion scores of ≥2, carcasses affected by ascariasis were almost 3.5 kg heavier than unaffected carcasses. Despite the low number of carcasses affected, this finding was not expected as pigs affected by ascariasis show a depressed growth rate (37) associated with a decrease in feed conversion efficiency (38). However, in support of our finding, Flesja and Ulvesaeter (36) reported that parasitic lesions, including “white spots” in the liver, occurred most frequently in the middle and heavy weight animals.

### Association between the Disease Lesions Responsible for Viscera Condemnation and VIS, Sex, and Tail Lesion Score

There were associations between the VIS and the likelihood of viscera being condemned for pneumonia, pleuropneumonia, and pericarditis at carcass level and for pneumonia at a batch level. These associations may reflect inconsistencies between VIS in the detection/identification and classification of disease lesions during meat inspection as previously reported by Elbers et al. (3). It is important to note that even though each VIS scored a relatively high number of farms (at least 10), the fact that some VIS may have scored farms with true differences in the occurrence of disease by chance cannot be dismissed.

Concerning tail lesion score, there was no relationship with viscera condemnation when analyzed at individual carcass level. However, condemnations due to pleurisy, pneumonia, and pleuropneumonia were associated with the overall tail lesion score at batch level. Schrøder-Petersen and Simonsen (17) reported that the lungs are the organs most easily affected by infection arising from tail biting. Similarly, Kritas and Morrison (21) reported an association between the severity of tail biting and enzootic pneumonia (EP) also at individual level. However, *Mycoplasma hyopneumoniae*, the bacteria responsible for EP, does not spread to the lungs via the blood (21). Hence, the pathogenesis of EP is unrelated to tail trauma and suggests that tail biting and EP may share similar risk factors. In contrast to the current study, Martínez et al. (39) found no association between pleuropneumonia and tail lesions. These authors suggested that the prevalence of pleuritis and pleuropneumonia may have been under reported as not all viscera could be inspected. This could have compromised a possible association between pleuropneumonia and tail lesions in their study.

The high prevalence of pleurisy, pneumonia, and pleuropneumonia in pigs originating from batches with higher tail lesion scores supports the association between poor health and poor welfare on pig farms. As mentioned above, it also reinforces the theory that, aside from providing an entry point for pathology, tail biting is associated with lung diseases because they share the same risk factors (23–25). It is thought that both conditions are elicited by stress (10), which suppresses the immune system and, therefore, contributes to an increased incidence of disease (40).

Abscesses are one of the main disease lesions responsible for carcass condemnation (16). Previous studies reported a close association between tail lesions and abscessation, both on the carcass (18, 20) and in the lungs (19, 21) or even with the presence of pyaemia (41). In the present study, the prevalence of lungs condemned for abscesses was surprisingly low (<1% of the study population), which contrasts with previous findings (21) and which may partially explain the absence of an association. Alternatively, the lack of relationship between tail lesions and lung abscesses could be explained by the fact that some tail lesions are healed before slaughter (39). There was also no association between viscera condemnation due to pericarditis or ascariasis and the severity of tail lesions, which suggests that these conditions do not share the same risk factors with tail biting. To our knowledge, no previous studies investigated this relationship.

### Methodological Issues

All condemnation data were recorded by the same researcher, on the basis of the decision of the acting temporary VI on the line. Some differences between our findings and those from other studies could be due to the different objectives of public health versus animal health monitoring (42). The effects found for VIS on viscera condemnation are representative of the reality of meat inspection in busy abattoirs and may have affected the associations with tail lesions.

It is likely that the findings reported in this study are an underestimation of tail biting, because animals that are severely affected may die or be culled on farm and are not sent for slaughter (22). Also, viscera are not removed for inspection from any carcasses that are entirely condemned (although this only represented 10 carcasses in the current study). Moreover, the prevalence of viscera condemnations was established on the basis of the decision of the acting VI on the line. Carcasses and viscera with pathological lesions resulting from disease or injury were partially or fully rejected on grounds of public health or consumer acceptability. It is possible that tail bitten pigs may have a higher rate of viscera pathologies, but not at a level that poses a threat to public health.

## Conclusion

This study showed a high prevalence of tail lesions and diseases associated with viscera condemnation. The relationship between overall tail lesion score and the lung diseases at batch level supports the relationship between poor health and poor welfare of pigs on farm and reinforces the potential inclusion of tail lesion scoring as part of the post-mortem meat inspection process for use as a pig health and welfare diagnostic tool. Recording animal health and welfare status during abattoir meat inspection and providing producers and their private veterinary practitioner with the results could support changes in management, feeding, or housing practices that will improve pig health, welfare, and performance thereby leading to economic benefits.

## Ethics Statement

This study was approved by the Teagasc Animal Ethics Committee (TAEC 24/2013).

## Author Contributions

DT, SH, LB, AH, NOC and SM contributed to the concept of the work. DT, LB and SH initiated, designed the study, and performed the experiment. DT and EM performed statistical analyses. DL, SM, EM, and LB interpreted data. DT and LB wrote the manuscript. SH, SM, NOC, AH, and EM contributed to the manuscript. All authors approved the final version of the manuscript.

## Conflict of Interest Statement

The authors declare that the research was conducted in the absence of any commercial or financial relationships that could be construed as a potential conflict of interest.
